# Examining the average scores of nursing teamwork subscales in an acute private medical ward

**DOI:** 10.1186/s12912-021-00609-z

**Published:** 2021-05-31

**Authors:** Martina Costello, Kylie Rusell, Tracey Coventry

**Affiliations:** 1 School of Nursing and Midwifery, Edith Cowan University, 270 Joondalup Drive, 6027 Joondalup, Western Australia; 2 School of Nursing & Midwifery, The University of Notre Dame Australia, 19 Mouat Street, 6160 Fremantle, Western Australia

**Keywords:** Nurs*, Nursing Teamwork Survey, Patient satisfaction, Staff satisfaction, Teamwork

## Abstract

**Background:**

Healthcare is delivered by multidisciplinary healthcare teams who rely on communication and effective teamwork to ensure safe patient care. Teamwork builds on employee cohesion and reduces medical and nursing errors, resulting in greater patient satisfaction and improved healthcare. Effective teamwork not only improves efficiency and patient safety but leads to a healthier and happier workplace, reducing burnout among healthcare professionals. The purpose of this paper is to describe the findings of a pilot project on an acute medical ward in Western Australia. The aim was to understand the participants perceived level of teamwork to support future work practices and ultimately patient care.

**Methods:**

This study used a descriptive survey research method to measure nursing teamwork in a clinical environment. The Nursing Teamwork Survey *(NTS)* measures the levels of nursing teamwork in acute healthcare facilities. Items for the NTS were generated on theoretical grounds, based on teamwork behaviours, offering a practical explanation of teamwork dynamics.

**Results:**

The survey incorporated five subscales. The response rate to the survey was 90 % (*n* = 45) with an overall average result on the survey being (*m* = 2.97) on a 0–4 Likert scale. The validated NTS has provided participants the opportunity to consider nursing teamwork with regards to their position and perceived responsibilities towards patients and team members.

**Conclusion:**

The findings highlight areas for consolidation and improvement in teamwork. Introducing teambuilding strategies and acting on results of this survey may support enhanced communication and teamwork influencing nursing care and patient outcomes. Findings recommend that activities to improve teamwork and ensuring teambuilding strategies are implemented to improve effective communication in an acute medical care setting would have significant impacts on staff satisfaction.

## Background

Contemporary research suggests that healthcare is delivered by multidisciplinary healthcare teams who rely on effective teamwork and communication to ensure safe patient care [1]. Effective teamwork builds on employee cohesion and reduces medical and nursing errors, resulting in greater patient satisfaction and improved healthcare [2]. Effective teamwork not only improves efficiency and patient safety but leads to a healthier and happier workplace, reducing burnout among healthcare professionals [1, 3].

The aim was to understand the participants perceived level of teamwork to support future work practices and ultimately patient care.

Ten national safety and quality in health service (NSQHS) standards were developed and endorsed by the Australian Commission on Safety and Quality in Health Care [ACSQH] (2018). The NSQHS standards aim to improve the quality of health service provision in Australia. The first standard relates to clinical governance for safety and quality [4]. This standard enables organisations to actively manage and improve the safety and quality of health care for patients.

Patient safety is enhanced when members within the multidisciplinary team engage in effective teamwork [5, 6]. Greco [7] describes the delivery process as multidisciplinary, requiring physicians, nurses, and allied health professionals to work in teams. It is these team performances that are crucial in providing safe patient care. Babiker et al., [5] reiterates that effective teamwork is globally recognized as an essential tool for constructing effective and patient-centred healthcare delivery systems which decrease medical errors and increase patient safety. Johnstone and Kanistsaki [8] reminisce that medical practice has traditionally focused on the individual physician as solely responsible for patient care. However, patients today are rarely looked after by just one health professional [9] and the medical system identifies that effective teamwork is critical for minimizing adverse events.

According to Barton, Bruce and Schreiber [10] nursing teams comprise the largest human resource delivering direct patient care in hospitals. WHO [9] reports there are 43.5 million health workers worldwide, with 20.7 million comprising of nurses. Nurses account for around 50 % of the healthcare workforce, therefore maximising their contribution to the health workforce is essential. Observational studies on teamwork behaviours related to high clinical performance, have identified patterns of communication, coordination, and leadership that support effective teamwork [11]. Kalisch et al., [3] suggest that nurses play an essential part in increasing patient access to safe quality care and identified that where nursing teamwork is rated as strong, there was a reduced reporting of missed patient care, fewer patient falls and a higher quality of work life impacting staff recruitment and retention. Manser, [12] investigated factors contributing to healthcare critical incidents and adverse events and found that the relationship between teamwork and patient safety is vital.

The success of an organisation can be measured on the loyalty of the employees and their job satisfaction [13]. Research has shown a direct correlation between staff satisfaction and patient satisfaction [14–16]. De Simone, Planta and Cicotto [17] also noted that work engagement, job satisfaction and self-efficacy influenced turnover intention and patient satisfaction. De Simone, Planta, and Cicotto [17] concluded that job satisfaction positively correlated with patient satisfaction, outlining that dissatisfied nurses who may be distracted from their patients, fail to provide holistic care which leads to a lower quality of nursing care. Bostan, Acuner and Yilmaz [18] described patient satisfaction as the reaction of healthcare users to different service aspects by comparing expectations of ideal care with their perception of quality of received care. Patient expectations and satisfaction with received nursing care, appear to be the greatest determinants for patient satisfaction regarding hospital care [19].

### Review of literature

A review of available literature between the years of 1991–2018, using the key words: Nurs*, Nursing Teamwork Survey, Patient satisfaction, Staff satisfaction, Teamwork and combined with the Boolean operators AND and NOT in CiNAHL, Medline, Cochrane and Joana Briggs data base was conducted. A review of the literature highlighted the importance of teamwork, and a health service’s responsibility to support its development and maintenance. Discussions at the health service identified no recent review or objective measurement of teamwork, which supported the research engagement.

Healthcare teams rely on effective teamwork and communication which ensures effective and safe patient care [14]. Effective teamwork has a range of benefits, which positively impact on; the quality and safety of patient care, employee satisfaction, patient satisfaction, staff retention and aids in the reduction of nursing and medical errors [11]. Teamwork builds cohesion among staff [9] and reduces the rate of burnout amongst healthcare professionals [10]. Effective teams are known to have a heightened sense of awareness, determining areas of interventions which leads to enhanced patient outcomes [10]. Therefore, the benefits of effective teamwork not only improve efficiency and patient safety but lead to a healthier and happier workplace [3].

Studies have shown that patient safety is enhanced when members, within the multidisciplinary team engage in effective teamwork [1, 3, 4, 10]. A team performance is critical in the provision of safe patient care and improved employee satisfaction [5, 7]. Babiker et al., [5] concludes that effective teamwork is a skill recognized globally, as an essential component which efficiently constructs patient-centred health care [12]. Effective teamwork is essential, reducing miscommunication and misunderstandings within a team’s roles and responsibilities [1, 3, 5, 10].

Clinically, nurses play an essential part maintaining patient therapeutic and professional relationships and increasing patient access to safe quality care [3]. Conversely Babiker et al., [5] discussed poor teamwork has a direct impact on adverse medical events, with increased evidence highlighting effective team functioning, has increased healthcare outcomes and patient safety. Kalisch et al., [3] identified hospital wards where nursing teamwork is rated as strong, reduced reporting of missed patient care and fewer patient falls and indicated a higher quality of work life impacting staff recruitment and retention.

Positive relationships are always beneficial, and the success of an organisation can be measured on the loyalty of the employees and their job satisfaction [13]. Research has shown a direct correlation between staff satisfaction and patient satisfaction within health care organisations [3, 5, 7, 13, 14]. Clinical studies report that healthcare workers who accomplish their assigned tasks, has positive effect upon employee satisfaction and patient satisfaction [14, 15], and enhanced job satisfaction among nursing staff, positively correlates with patient satisfaction. Dissatisfied nurses fail to provide holistic care which leads to a lower quality of nursing care [14, 15, 20]. Swain [15] suggested that nursing employee job satisfaction is an important aspect for quality care, it is vital to support the well-being of staff due to the potential to improve patients’ perceptions of quality care. Bostan et al., [18] described patient satisfaction as the reaction of healthcare users to different service aspects by comparing expectations of ideal care with their perception of quality of received care. Furthermore, Bostan et al., [18] described how in theory patient satisfaction is connected to nursing care. The best determinants of overall satisfaction with hospital care appear to be patient expectations and satisfaction with nursing care (Abramowitz et al., [19]).

#### Aim

The purpose of this study was to describe the findings of nursing teamwork subscales in an acute private medical ward in Western Australia. Understanding participants perceived level of teamwork may support future work practices and ultimately patient care.

## Method

### Research Design

This project used a quantitative approach. Quantitative research aims to measure, describe and, or compare aspects of the world in terms of numerical data. The quantitative research approach facilitates the development of statistical information which can be used to describe and potentially make generalisations about the relevant population [21].

This study utilised a quantitative descriptive design research method to measure nursing teamwork in a clinical environment. A cross-sectional descriptive design using the NTS tool was used to investigate RNs perceptions of teamwork within a medical ward in one health care facility. The NTS has been used in a number of international studies measuring nursing teamwork [22, 23]. This design provided data from a convenience sample of registered nurses working within an Australian healthcare organisation. Survey research is defined as “the collection of information from a sample of individuals through their responses to questions” [21]. This method was chosen because it provides the practical convenience of obtaining information from a group of nursing staff over a short period of time by one researcher at minimal cost [20]. This was a time restricted project study seeking to describe nurses’ perceptions of teamwork on an acute medical ward, there was forecasted targeting of a specific sample size. In the facility there is on average thirty nurses working over a 24-hour period. The Nursing Teamwork Survey *(NTS)* was developed by Kalisch, Lee and Salas [24] to measure the levels of nursing teamwork in acute healthcare facilities to assist in identifying the level of nursing teamwork to enable team quality improvement. Informed by the Salas teamwork model [25] items for the NTS were formed on theoretical grounds. Teamwork behaviours was the bases of Salas theory [25], which offered a practical description of teamwork dynamics. The *NTS* content validity index was 91.2 % (on the final version of the NTS) based on the re-view of the expert panels’ assessment of the relevance and clarity of the NTS [25].

### Site and Population

This study was conducted in a private healthcare group in Western Australia. The healthcare organisation incorporates three exclusively private hospitals and two Public Private Partnership (PPP) hospitals. The organisation cares for both public and private patients and has a total of 722 licensed beds and bays. The facility is one of the largest health services in Western Australia, treating more than 73,000 inpatients annually [26].

The study site was an acute medical ward within one of the PPP hospitals. This hospital was chosen for the study due to its location within the metropolitan area and the researcher’s experience, knowledge and familiarity with systems and employees within the facility. The population under study were full time and part time nursing employees (*n* = 50) employed on a medical ward. The population included registered nurses, enrolled nurses (EN), clinical nurses and a clinical nurse manager. Participants, self-selected to take part in the study, through voluntary completion of the survey. Exclusion criteria included assistants in nursing and other professions within the multidisciplinary team. Using Yamane’s equation [27] for simplified formula for proportions the sample size required was 45 to describe patterns. The confidence level was 95 % and *p* = 0.5 are assumed and the acceptable sampling error was 0.05.

### Data collection and analysis

The researcher (enrolled in a Master of Nursing, coursework) was responsible for the collection and analysis of data with the support of two research supervisors. Data was collected through an anonymous, paper-based NTS survey which was made conveniently available to all staff on a medical ward. The survey comprised of a series of Likert scale response items. Convenient deployment of surveys allowed for rapid data collection and cost-effectiveness. However, disadvantages may have included limited sampling and respondent cooperation. The NTS was accessible via printed copies with a cover letter inclusive of survey instructions and the participant information sheet that clearly stated that completion of the NTS was considered as consent Participants placed the completed survey into a provided envelope, and into a locked box for anonymous submission over a four-day period. Data was exported, cleaned and coded, prior to being imported into an excel work sheet. Descriptive statistics were generated. Results are presented below in terms of number of participants, mean and standard deviation. Demographics are presented first, followed by analysis of the participants’ responses to the *NTS* tool.

### Ethical considerations

 Participants were provided with an information sheet which clarified completion of the NTS was considered as consent. Participants were provided with a participant information sheet. This approach was approved by the Human Research Ethics Committee at Ramsay Health Care Western Australia/South Australia (approval #1637) and the University of Notre Dame Australia (approval #310,816).

## Findings

There are two NTS available; a 45-question (long version) NTS which contains questions regarding demographic characteristics of the participants, including education level, gender, age and job title. The ‘long survey’ also contains employment related items such as shift allocations and years of experience, levels of teamwork on the unit, staffing adequacy, fulfilment of current role and position. The 33 question NTS short version is designed to specifically evaluate nursing teamwork perceptions in individual wards in acute care hospital settings [24]. The survey does not include demographic data to ensure confidentiality with potentially small sample sizes.

The Thirty-three items of the NTS measure the descriptors of teamwork and are divided into five subscales based on Salas’ ‘Big Five’ framework of teamwork [24]. The NTS uses a 5-point Likert-scale as follows: Rarely = 0; 25 % of the time = 1; 50 % of the time = 2; 75 % of the time = 3; Always = 4. The maximum score for each group is 132 which would demonstrate perfect teamwork.

**The five subscales are defined** [24].

1. Trust: Team members trust each other enough to communicate ideas and information and value, seek and give each other constructive feedback. Confidence that team members will demonstrate ways which promote the aims of the team.
2. Team orientation: The team works together to improve each other’s weaknesses efficiently and effectively. Cohesiveness and the group’s awareness of itself as a team.
3. Backup: Team members willingly aid and help one another when they recognise someone is busy or overloaded with work. Helping one another with their tasks and responsibilities.
4. Shared mental model: All team members understand their role and responsibilities and thus respectively work together to achieve a quality work outcome.
5. Team leadership: Charge nurses or managers adequately monitor, distribute and balance the workload of the nurses. Structure, direction, and support provided by the formal leader on the part of team members.

**NTS short questions** [24].

1.  All team members understand what their responsibilities are throughout the shift.
2.  The nurses who serve as charge nurses or team leaders monitor the progress of the staff members throughout the shift.
3.  Team members frequently know when another team member needs assistance before that person asks for it.
4.  Team members communicate clearly what their expectations are of others.
5.  Mistakes and annoying behavior of teammates are not ignored but are discussed with the team member.
6.  When changes in the workload occur during the shift (admissions, discharges, patients’ problems etc.), a plan is made to deal with these changes.
7.  Team members know that other members of their team follow through on their commitment.
8.  The nurses who serve as charge nurses or team leaders balance workload within the team.
9.  My team believes that to do a quality job, all of the members need to work together.
10.  The shift change reports contain the information needed to care for the patients.
11.  Team members usually return from breaks on time.
12.  Team members respect one another.
13.  When a team member points out to another team member an area for improvement, the response is never defensive.
14.  Team members are aware of the strengths and weaknesses of other team members they work with most often.
15.  If the staff on one shift is unable to complete their work, the staff on the on-coming shift do not complain about it.
16.  Staff members with strong personalities do not dominate the decisions of the team.
17.  Most team members tend to deal with conflict rather than avoid it.
18.  Nursing assistants and nurses work well together as a team.
19.  The nurses who serve as charge nurses or team leaders are available and willing to assist team members throughout the shift.
20.  Team members notice when a member is falling behind in their work.
21.  When the workload becomes extremely heavy, team member’s pitch in and work together to get the work done.
22.  Feedback from team members is often helpful rather than judgmental.
23.  My team readily engages in changes in order to make improvements and new methods of practice.
24.  Team member information with each other.
25.  Team members clarify with one another what was said to be sure that what was heard is the same as the intended message.
26.  Team members work together to achieve the total work of the team.
27.  The nurses who serve as charge nurses or team leaders give clear and relevant directions as to what needs to be done and how to do it.
28.  Within our team, members are able to keep an eye out for each other without falling behind in our own individual work.
29.  Team members understand the role and responsibilities of each other.
30.  Team members willingly respond to patients other than their own when other team members are busy or overloaded.
31.  Team member’s value, seek and give each other constructive feedback.
32.  When someone does not report to work or someone is pulled to another unit, we reallocate responsibilities fairly among the remaining team members.
33.  Team members trust each other.

**Fig. 1:**
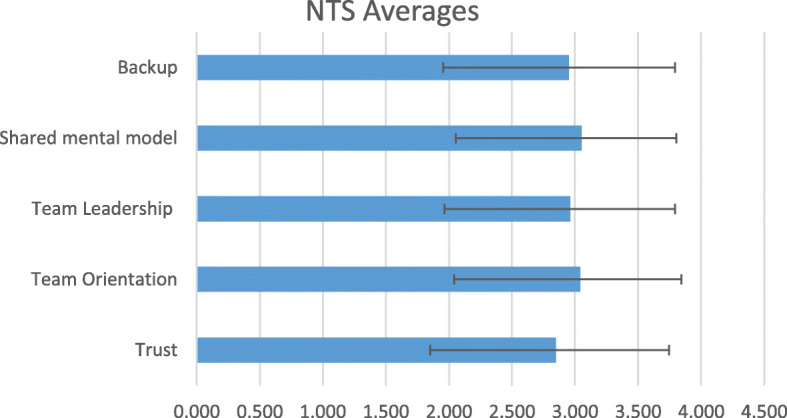
Comparison of the five subscales. Scores illustrated are means ± standard deviation

Participation was 90 % (*n =* 45). The mean value for ward result across all questions 2.97 ± 0.82. The total ward average survey score, across the five themes, is outlined in Fig. 1, which indicates overall participants had a positive engagement with teamwork.

### Trust (questions 1–7)

Question 1 scored the highest total score of 152/180 (84.4 %), with a mean score of 3.4 ± 0.64 whilst, Question 5, scored the lowest, with a score of 101/180 = 56.1 % with a mean score of 2.2 ± 1.19. The Combined Trust Factor Score was 898 out of a possible 1260 points = 71.2 % with an average mean score of 2.85 ± 0.89, this was the lowest across the survey.

Trust within the NTS measures the ability for teams to be trusting enough of one another in order to communicate effectively, discussing ideas where the team values opinions and facilitates constructive feedback. Question 5 yielded the lowest score with 56 % of the nurses surveyed believing that mistakes and annoying behaviours of teammates are ignored and not discussed.

### Team orientation (questions 8–16)

Team orientation had an overall mean score of 3.04 ± 0.80. This may indicate a positive culture of mentorship among nursing staff. Within the nursing profession there is a variation in levels of knowledge and experience. Having a mixed skill set in the team comprising of different levels of knowledge and experience is a positive, helping nursing staff to develop skills. Question eleven scored the highest with 86 % of staff agreeing that team members usually return from breaks on time. A reason for this could be staff respecting their colleagues, additionally, adequate staffing, allows nurses to feel confident when taking break’s knowing their patients are being cared for.

Question 16; *“Staff members with strong personalities do not dominate the decisions of the team”* had 109 of 180 nurses agree (60.5 %), with an average mean score of 2.4 ± 1.02. This result may be due to how team meetings are conducted and how the team respectfully communicates.

The total score for Team orientation was 1232/1620 equating to 76 % with a mean score of 3.04 ± 0.80.

### Backup (Questions 17–22)

Backup received a mean score of 2.96 ± 0.83, 74 % of participants believed that team members willingly aid and help one another when they recognise someone is busy. Due to the open aspects of the ward teams can visually see one another to gauge if their colleague is overworked. The manager on this ward encourage teams to work and support each other. The shift coordinator also takes a patient load if acuity on the ward is high and staff are overworked. Question 17 “*Most team members tend to deal with conflict rather than avoid it*”. A majority of 63 % of the population believed that this was correct, with a score of 144/180, and a mean score of 2.5 ± 0.85.

Question 18 “*nursing assistants and nurses work well together as a team*”. 144/180 participants (80 %) with an overall average equalling a mean of 3.2 ± 0.77 nurses surveyed agreeing. This may be due to RNs and nursing assistants communicating effectively when providing care.

The total score for Backup was 800/1080 equating to 74 % with a mean of 2.96 ± 0.83.

### Shared mental model (questions 23–29)

The Shared mental model scored highest in this study with a mean score of 3.05 ± 0.74. This may be because nurse managers optimize and utilise each nurse’s scope of practice. The ability to allocate nurses according to patient acuity may reinforce team functioning and response to patient’s needs. Nurses also receive a comprehensive handover of patients, which enables accurate shift expectations, and supports planning.

Question 26 *“team members working together to achieve the total work of the team*” results were 143/180 (79.4 %) with a mean of 3.2 ± 0.70. Participants believed that nurses within their team worked together to achieve goals of care. The nursing skill mix and positive culture among staff may contribute to this.

Question 23 related to how team members readily engage in change to make improvements of practice. The score of 128/180 = 71.1 % with a mean score of 2.9 ± 0.79 of nurses believed that change leads to improvements and new methods of practice. This may have benefited from the management structure and staff respecting policy and procedures.

The total was 959/1260 = 76.1 % which had the overall highest average and mean score of 3.05 ± 0.70.

### Team leadership (Questions 30–34)

Team leadership result resulted in a mean of 2.96 ± 0.83, which is the same average as Backup. Team leadership measures if senior nurses/managers adequately monitor, distribute and balance the workload of nurses. This result may indicate the nurse manager and clinical nurse’s engagement with ensuring appropriate workload and staffing levels. 81 % of participants agreed with question 33 “Team members trust each other”. In comparison, 73 % agreed with question 30, “team members willingly respond to patients other than their own when other team members are busy or overloaded”.

The total 534/720 = 74.1 % with a mean of 2.96 ± 0.83.

## Discussion

This study evaluated a self-report of nursing teamwork and interactions within a clinical environment. Using the NTS provided data about how nurses regard their positions, responsibilities, team members and the ward as a whole.

A comparison of results could not be achieved, as no published literature on the tool in an acute medical ward could be identified. However, a comparison to other hospitals was possible.

Kaiser and Westers [22], completed the survey in five departments including paediatrics, emergency, surgical, ICU and rehabilitation, noted that the Rehabilitation ward had the highest levels of total teamwork (*m* = 3.840) and continuing care (long term care) had significantly lower levels (*m* = 3.288). In a similar Australian study by Chapman, Rahman, Courtney and Chalmers [28] RNs and ENs working within one public sector health network in ICU, surgical, medical, and specialist wards including ED, coronary care, and rehabilitation units at four hospitals, recognised that nursing staff content with the level of teamwork on their unit scored significantly higher in the NTS overall (*m* = 3.70) than the nursing staff who were dissatisfied (*m* = 2.95) with the medical ward scoring (*m* = 3.46 [28]. Furthermore, a study by Kalisch et al., [3] provided results from 50 medical-surgical, intermediate, intensive care, and rehabilitation units in four hospitals located in America. This study achieved a higher mean satisfaction with only one site.

Trust measured if participants trusted in one another to communicate ideas and information and to value, to seek, and to give each other constructive feedback, in conjunction with shift responsibilities [24]. Trust scored the lowest with a mean of 2.85 ± 0.89. Question five yielded the lowest score of the survey with 56 %, these nurses believed that mistakes and annoying behaviours of teammates are ignored. According to Grubaugh and Flynn [6] conflit due to insecurity or lack of competence in managing stressful, conflict situations impedes healthy communication and escalates stress among team members. Brinkert [29] also believes that a more effective approach to conflict, such as competition, accommodation, or collaboration have been found to facilitate teamwork and interprofessional relationships. Bochatay [30] discussed the importance of supportive leadership which contributed to positive conflict management outcomes. To be effective, conflict resolution requires nurse leaders to have knowledge and skills in order to select the best approach for conflict situations. In addition, teams are required to take one another’s behaviour into account during group interactions. In order to work cohesively and cooperatively, team members can achieve this by predicting and anticipating each other’s needs through common understandings of the environment and expectations of performance [25].

A shared belief that team members will perform their roles and protect the interests of their teammates is essential [25]. For teams to freely communicate information, the team must preserve a degree of mutual trust [31]. The key to a better healthcare team includes organization and clearly identified roles. This results in a team with improved confidence in their specific skills, clarity in their role positions and team leadership [32]. According to Ramanujam and Rousseau [33] healthcare organisations operate with numerous conflicting demands and handle challenging daily tasks. Consequently, attention needs to focus on coordination, role allocation and shared responsibility of staff [34]. Ramanujam and Rousseau [33] recommend clear goal setting, feedback, service redesign and positive involvement of staff.

The Backup subscale scored markedly low for ‘Within our teams, members are able to keep an eye out for each other without falling behind in our own individual work’. Kalisch et al., [3] suggested that demands for patient care vary from moment to moment and nurses cannot provide all planned care by themselves. When teamwork is present, staff perceive the work as “ours” and not the particular staff members work who it has been assigned. This leads to nurses moving to assist their colleague and providing a backup for one another up. Additionally, Grubaugh and Flynn [6] noted that willingness to assist team members, known as team backup, has been theoretically and empirically identified as a core indicator of effective teamwork.

Shared mental model scored highest in this study. The items measure nurses understanding of their role and responsibilities, to achieve quality work outcomes [24]. This result indicated that team members were clear on the responsibilities and tasks expected of themselves and others. They felt they worked well together and respected and valued each other. The shared mental model includes involvement, information sharing, strategizing, and participating in goal setting, which are required for effective teams [25]. Individuals also have a clear understanding of their roles in the task, of the resources available, and of their teammates’ capabilities (i.e., task related competencies, preferences). Information sharing and a willingness to admit mistakes and accept feedback is essential. Teams who take regular time out to evaluate and reflect on performance, increase efficiency yielding better outcomes and exhibit higher levels of innovation required for teamwork [35].

Graen, Canedo and Grace [36] discussed the quality of relationships between employees and their supervisor. This is known as the Leader–member exchange (LMX) theory. In the nursing profession, the application of the LMX theory shows positive outcomes in affective commitment, job satisfaction, and reduced intention to leave. The LMX relationship comprises of four measurements: contribution; the degree of work-related effort performed; loyalty, the exhibition of public support in the leader–member relationship; affect, the interpersonal liking in the leader–member dyad; and professional respect, the degree to which each member of the dyad has built a credible reputation [34]. In the nursing context, researchers have found that LMX is positively related to positive staff perceptions of safety behaviours, job satisfaction, extra‐role behaviour, and trust in supervisor, organizational commitment and knowledge sharing [35].

Employers have different types of relationships with employees within their organisation. Graen et al., [33] explained that employees with high quality LMX demonstrated competence, trustworthiness and motivation, they also held greater responsibility and received superior attention and additional support from their employers. This favoured treatment was likely to be reciprocated and such employees tended to go above and beyond the duties. Conversely, low-quality LMX individuals have a contractual relationship with their employer providing only what is set out in a contractual clause [33]. Furthermore, Graen et al., [33] also discussed the quality of relationships between employees and their supervisor in which subordinates with high quality LMX reciprocate desirable qualities. Implementation of the LMX theory could therefore provide significant enhancements to the quality of teamwork relationships.

Effective teamwork is widely recognised and well known as an important factor in staff satisfaction in health care environments contributing to high quality patient care [37] (Ramsay Health Care, 2018). In addition, safe and quality care of the patient can also be attributed to effective teamwork [3]. Effective teams can offer greater flexibility, efficiency, and creativeness, more so than one individual. Together teams can provide superior multifaceted solutions to organizational complications [38]. However, teams are not easily implemented, a team of skilled participants does not guarantee success, building successful teams requires dedication, understanding and compliance [36].

Results of the study indicate a need to enhance nursing teamwork at the study site. Several evidence-based approaches to advance, improve and ensure successful team training can include alignment of organisational goals, greater depth of organisational support, role modelling by ward leaders, improving culture and environment, targeted resources and time to apply new skills and knowledge and frequent evaluation of progress [39, 40].

### Limitations

The authors acknowledge that a limitation to this study is that it was conducted on one ward within a large hospital, and therefore these findings may not be reliable for other work areas. A convenience sample of nurses who self-selected to participate may also be a limitation; the results are based on responses to the *NTS* rather than observations of team behaviours’ which may be influenced by the perceptions of the employee surveyed.

### Implications

Results of the present study highlight the need to enhance nursing teamwork. Salas et al. [41] recommends several evidence-based strategies which enables a manager to enhance, sustain and develop effective team training: alignment of safety aims and team training objectives with organizational goals; boost participation of frontline leaders; provide organizational support; ensure suitable preparation of staff and the training environment; availability of adequate training resources and sufficient time commitments; explicit facilitation to ensure application of required teamwork skills and effective measurements of the success of the team training programs.

Nurse managers employ strategies to support teamwork interventions, ensuring ongoing measurements of satisfaction and the effect of such strategies are reflected on and considered.

### Recommendations

Additional research is essential to determine the accuracy of nurse’s perceptions on relationships within their team; Communication between team members can enhance relationships [42]. Results from the *NTS* may always be different for every workplace due to a diverse range of leadership styles and culture. Recommendations are that leaders should know their team and seek out this information to ensure that strategies are meaningful and relevant to their workplace. Positive reinforcement of the benefits of effective communication is encouraged. Further studies, including a subsequent second survey, with a larger sample size across the health care facility, will allow a comparison to the current study.

## Conclusions

Contemporary healthcare is delivered by multidisciplinary, distributed healthcare teams who rely on effective teamwork and communication to ensure effective and safe patient care. Teamwork leads to higher staff job satisfaction, enhanced patient safety, improved quality of care, and greater patient satisfaction. Such benefits of effective teamwork not only improve efficiency but lead to a healthier and happier workplace. The findings of this study have expanded the understanding of how nursing staff perceive teamwork, in regards to their positions, responsibilities, team members and the ward as a whole at the study site. The main aim of this study was to highlight areas of teamwork strength and deficit to facilitate the implementation of strategies with subsequent evaluation. Introducing teambuilding strategies and acting on the results of this survey may also provide effective support to help improve communication and teamwork which will ultimately improve the quality of nursing care and patient outcomes.

## Data Availability

The datasets used and/or analysed during the current study are available from the corresponding author on reasonable request.

## Abbreviations

NSQHS: national safety and quality in health service
ACSQH: Australian Commission on Safety and Quality in Health Care
WHO: World Health Organisation
NTS: Nursing Teamwork Survey
PPP: Public Private Partnership
EN: Enrolled nurses
LMX: Leader–member exchange
(m): Mean
